# Impact of specific functional groups in flavonoids on the modulation of platelet activation

**DOI:** 10.1038/s41598-018-27809-z

**Published:** 2018-06-22

**Authors:** Divyashree Ravishankar, Maryam Salamah, Angela Akimbaev, Harry F. Williams, Dina A. I. Albadawi, Rajendran Vaiyapuri, Francesca Greco, Helen M. I. Osborn, Sakthivel Vaiyapuri

**Affiliations:** 1 School of Pharmacy, University of Reading, Reading, UK; 2 School of Pharmacy, University of Reading Malaysia, Johor, Malaysia

## Abstract

Flavonoids exert innumerable beneficial effects on cardiovascular health including the reduction of platelet activation, and thereby, thrombosis. Hence, flavonoids are deemed to be a molecular template for the design of novel therapeutic agents for various diseases including thrombotic conditions. However, the structure-activity relationships of flavonoids with platelets is not fully understood. Therefore, this study aims to advance the current knowledge on structure-activity relationships of flavonoids through a systematic analysis of structurally-related flavones. Here, we investigated a panel of 16 synthetic flavones containing hydroxy or methoxy groups at C-7,8 positions on the A-ring, with a phenyl group or its bioisosteres as the B-ring, along with their thio analogues possessing a sulfur molecule at the 4^th^ carbon position of the C-ring. The antiplatelet efficacies of these compounds were analysed using human isolated platelets upon activation with cross-linked collagen-related peptide by optical aggregometry. The results demonstrate that the hydroxyl groups in flavonoids are important for optimum platelet inhibitory activities. In addition, the 4-C=O and B ring phenyl groups are less critical for the antiplatelet activity of these flavonoids. This structure-activity relationship of flavonoids with the modulation of platelet function may guide the design, optimisation and development of flavonoid scaffolds as antiplatelet agents.

## Introduction

Platelets are small circulating blood cells that play pivotal roles in the regulation of haemostasis upon vascular injury through blood clotting^1,2^. However, unnecessary activation of platelets within the vasculature leads to pathological conditions such as thrombosis, which results in blockage or reduction of blood flow to major organs including heart and brain instigating heart attack and stroke, respectively^3,4^. The currently used therapeutic options that involve the use of antiplatelet drugs such as clopidogrel, aspirin, and prasugrel are often linked with adverse side effects such as bleeding and are ineffective in some patients^5–10^. As cardiovascular diseases remain the leading cause of death worldwide^11^, the development of improved therapeutic strategies to prevent and treat thrombotic diseases remains a pressing priority.

Flavonoids, a group of polyphenolic plant metabolites, have been widely demonstrated to possess beneficial effects in the prevention of cardiovascular diseases^12–14^. Epidemiological and clinical studies have established a prominent link between the regular consumption of dietary flavonoids and decreased incidences of cardiovascular diseases or their risk markers^15–20^. Flavonoids have also been recognised as modulators of platelet function and their inhibitory activities can be attributed to their ability to inhibit reactive oxygen species (ROS) production^21,22^, modify cytoskeletal proteins such as actin and tubulin that mediate degranulation^23,24^, and to inhibit various kinases^25–29^ and receptors^30,31^ that play numerous roles in the regulation of platelet activation and thrombosis. The pharmacological potential of flavonoids is strongly related to their molecular structure, hence, the identification of key structural elements that are prerequisites for antiplatelet activity has provoked considerable interest in the area of drug discovery^32,33^. With a view to develop flavonoids as potential anti-thrombotic agents, some studies have been carried out to identify the key structural features governing the antiplatelet activity of flavonoids^34^. However, the current knowledge of structure-activity relationships (SARs) has mainly resulted from analyses involving different subclasses of natural flavonoids^32,34^. In order to translate flavonoids into potential molecular templates for drug design, a better understanding of the SAR of flavonoids, and a careful comparison of substitution pattern within a flavonoid subclass is necessary. Hence, to gain greater insights into the SAR of flavonoids with human platelets, this study has focused on assessing the effect of methoxylation, 4-C=S substitution, and different B-ring substitutions using a series of 16 synthetic flavones on the antiplatelet activities.

## Materials and Methods

### Flavones

The synthetic flavones used in this study are grouped into four main classes namely; hydroxy flavones with free –OH and 4-C=O, hydroxy 4-thioflavones with free –OH and 4-C=S, methoxy flavones with –OMe and 4-C=O and methoxy 4-thioflavones with–OMe and 4-C=S^35^. The hydroxy flavones include 7,8-dihydroxy-2-phenyl-4*H*-chromen-4-one (F-1), 7,8-dihydroxy-2(thiophen-2-yl)-4*H*-chromen-4-one (F-2), 2-(furan-2-yl)-7-8,dihydroxy-4*H*-chromen-4-one (F-3) and 7,8-dihydroxy-2(pyridine-3yl)-4*H*-chromen-4-one (F-4). The hydroxy 4-thioflavones include 7,8-dihydroxy-2-phenyl-4*H*-chromen-4-thione (TF-1), 7,8-dihydroxy-2(thiophen-2-yl)-4*H*-chromen-4-thione (TF-2), 2-(furan-2-yl)-7-8,dihydroxy-4*H*-chromen-4-thione (TF-3) and 7,8-dihydroxy-2(pyridine-3yl)-4*H*-chromen-4-thione (TF-4). The methoxy flavones include 7,8-dimethoxy-2-phenyl-4*H*-chromen-4-one (CYC-1), 7,8-dimethoxy-2-(thiophen-2-yl)-4*H*-chromen-4 (CYC-2), 2-(furan-2-yl)-4*H*-chromen-4-one (CYC-3) and 7,8-dimethoxy-2-(pyridine-3-yl)-4*H*-chromen-4-one (CYC-4). The methoxy 4-thioflavones include 7,8-dimethoxy-2-phenyl-4*H*-chromen-4-thione (TCYC-1), 7,8-dimethoxy-2-(thiophen-2-yl)-4*H*-chromen-4-thione (TCYC-2), 2-(furan-2-yl)-7,8-dimethoxy-4*H*-chromen-4-thione (TCYC-3) and 7,8-dimethoxy-2-(pyridine-3-yl)-4*H*-chromen-4-thione (TCYC-4). Stock solutions of these flavones were prepared in dimethyl sulfoxide (DMSO) (100%) at 10 mg/mL concentration and the final required test concentrations were obtained by appropriately diluting these stocks. The final concentration of DMSO in platelets was maintained at 0.1% (v/v), which did not affect their function.

### Human blood collection

All the experiments in this study were conducted in line with the appropriate institutional and national guidelines and regulations. Human blood was collected by venepuncture from aspirin free, healthy volunteers into vacutainers containing 3.2% (v/v) citrate after obtaining their informed consent. The procedures and consent forms used in this study were approved by the University of Reading Research Ethics Committee.

### Preparation of human isolated platelets

Human isolated platelets were prepared by adding 7.5 mL of ACD [(acid citrate dextrose) (20 g/L glucose, 25 g/L sodium citrate and 15 g/L citric acid)] to 50 mL of blood prior to centrifugation at 100 g for 20 minutes at room temperature. The Platelet-Rich Plasma (PRP) was removed using wide bore pipette tips and mixed with 3 mL of ACD and centrifuged at 1400 g for 10 minutes at room temperature. The resulting platelet pellet was resuspended in modified Tyrodes-HEPES buffer (2.9 mM KCl, 134 mM NaCl, 0.34 mM Na_2_HPO_4_.12H_2_O, 1 mM MgCl_2_, 12 mM NaHCO_3_, 20 mM HEPES, pH 7.3) and centrifuged again at 1400 g for 10 minutes^28^. The final platelet pellet obtained was suspended in modified Tyrodes-HEPES buffer at a density of 4 × 10^8^ cells/mL for aggregation assays and rested at 30 °C for 30 minutes before using in platelet functional assays.

### Platelet aggregation assays

Platelet aggregation was performed using a platelet glycoprotein VI (GPVI)-selective agonist, CRP-XL (obtained from Professor Richard Farndale, University of Cambridge) in the presence or absence of a vehicle control [0.1% (v/v) DMSO] or different concentrations of flavone derivatives by optical aggregometry. Human isolated platelets (267 µL) taken in siliconised cuvettes were incubated with 3 µL of a vehicle control or various concentrations of flavone derivatives for 5 minutes at 37 °C. Following the incubation, 30 µL of CRP-XL (0.5 µg/mL) was added to platelets and the level of aggregation was measured for 5 minutes at 37 °C under constant stirring (1200 rpm). Data were analysed by calculating the percentage of maximum platelet aggregation at 5 minutes, and the level of aggregation obtained with the vehicle control was considered as 100% to quantify the impact of flavones on platelets.

### Lactate dehydrogenase assay

The lactate dehydrogenase (LDH) assay was performed using a LDH Cytotoxicity Assay Kit (Pierce, Thermo Fisher) according to the manufacturer’s instructions. Briefly, to 50 µL of human isolated platelets, 1 µL of a positive control [1% (v/v) Triton-X 100, provided in the kit] or a vehicle control [0.1% (v/v) DMSO] or various concentrations of flavone derivatives were added and incubated for 30 minutes at 37 °C. Then, 25 µL of the reaction mixture (provided in the kit) were added to the platelets and further incubated for 30 minutes in dark. Finally, the reaction was stopped by adding 25 µL of stop solution (provided in the kit). The absorbance was measured at 490 and 650 nm using a Fluostar Optima spectrofluorimeter (BMG Labtech, Germany). The level of LDH released with the positive control was considered as 100% to quantify the LDH release in flavone-treated samples.

### Statistical analysis

The statistical significance between the vehicle controls and flavones-treated platelet samples was analysed by one-way ANOVA followed by Bonferroni *post-hoc* test. All the statistical analyses were performed using GraphPad Prism 7 (GraphPad Software Inc., USA).

## Results

**Figure 1:**
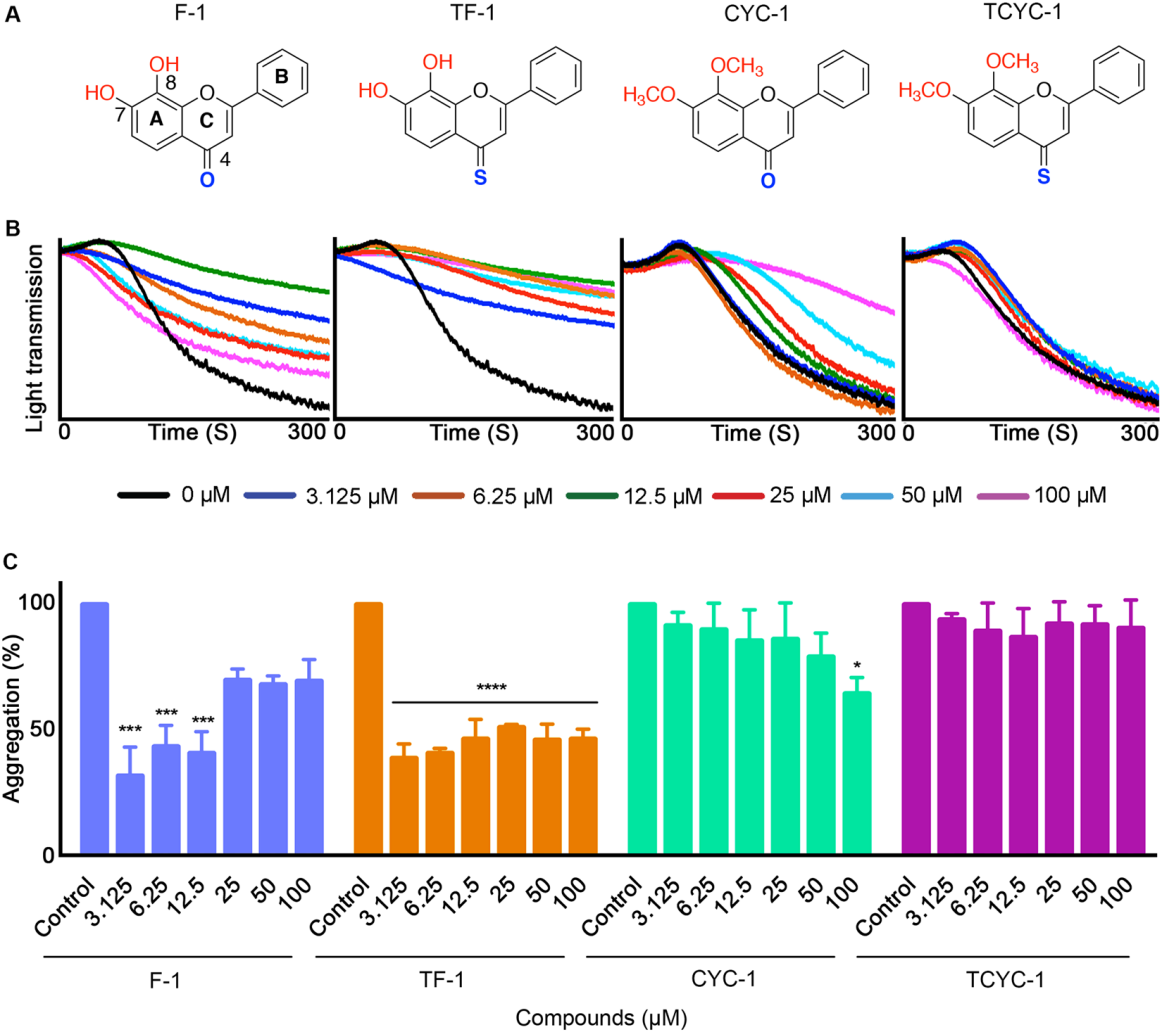
Effect of flavones with phenyl B-ring on human platelet activation. (**A**) Chemical structures of the flavones, F-1, TF-1, CYC-1 and TCYC-1. (**B**) Representative traces displaying the level of aggregation obtained when human isolated platelets were treated (for 5 minutes) with a vehicle control [0.1% (v/v) DMSO] or various concentrations of flavones, F-1, TF-1, CYC-1 and TCYC-1 (3.125–100 μM) and 0.5 μg/mL CRP-XL. (**C**) Bar graphs show the percentage of aggregation obtained in the presence and absence of different concentrations of flavones, F-1, TF-1, CYC-1 and TCYC-1. The data were normalised by considering the maximum aggregation observed for the vehicle control at 5 minutes as 100%, and the level of inhibition in flavone and its derivatives-treated platelet samples was calculated accordingly. Cumulative data denote mean ± S.D. (n = 3). The *p* values displayed (*p < 0.05, ***p < 0.001 and ****p < 0.001) are as analysed by one-way ANOVA using Graphpad Prism.

**Figure 2:**
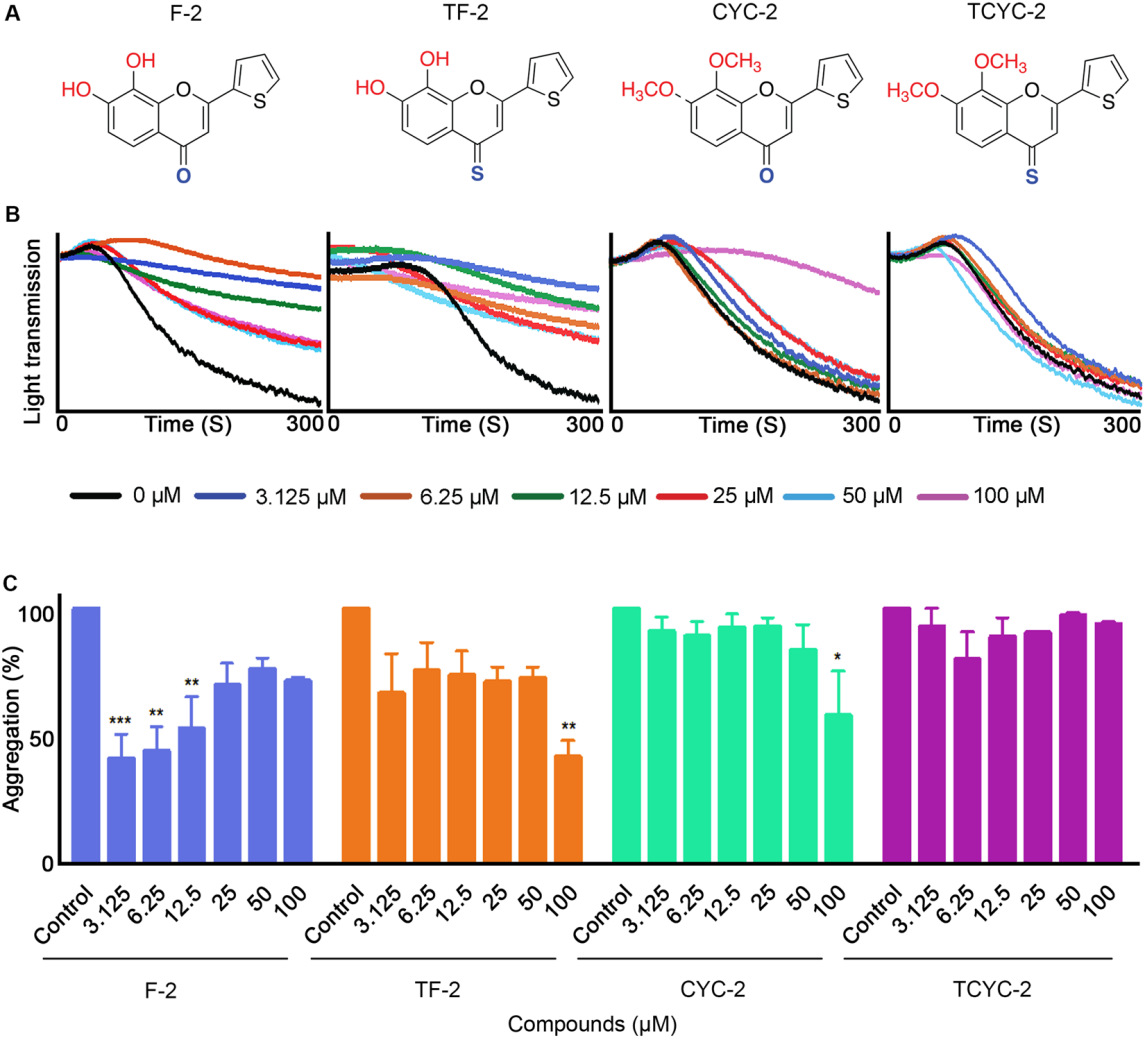
Effect of flavones with thiofuran B-ring on human platelet activation. (**A**) Chemical structures of the flavones, F-2, TF-2, CYC-2 and TCYC-2. (**B**) Representative traces showing the level of aggregation obtained when human isolated platelets were treated (for 5 minutes) with a vehicle control [0.1% (v/v) DMSO] or diverse concentrations of flavones, F-2, TF-2, CYC-2 and TCYC-2 (3.125–100 μM) and 0.5 μg/mL CRP-XL. (**C**) Bar graphs show the percentage of aggregation obtained in the presence and absence of various concentrations of flavones, F-2, TF-2, CYC-2 and TCYC-2. The data were normalised by considering the maximum aggregation observed for the vehicle control at 5 minutes as 100%, and the level of inhibition in flavone and its derivatives-treated platelet samples was calculated accordingly. Cumulative data denote mean ± S.D. (n = 3). The *p* values displayed (*p < 0.05, **p < 0.01 and ***p < 0.001) are as analysed by one-way ANOVA using Graphpad Prism.

**Figure 3:**
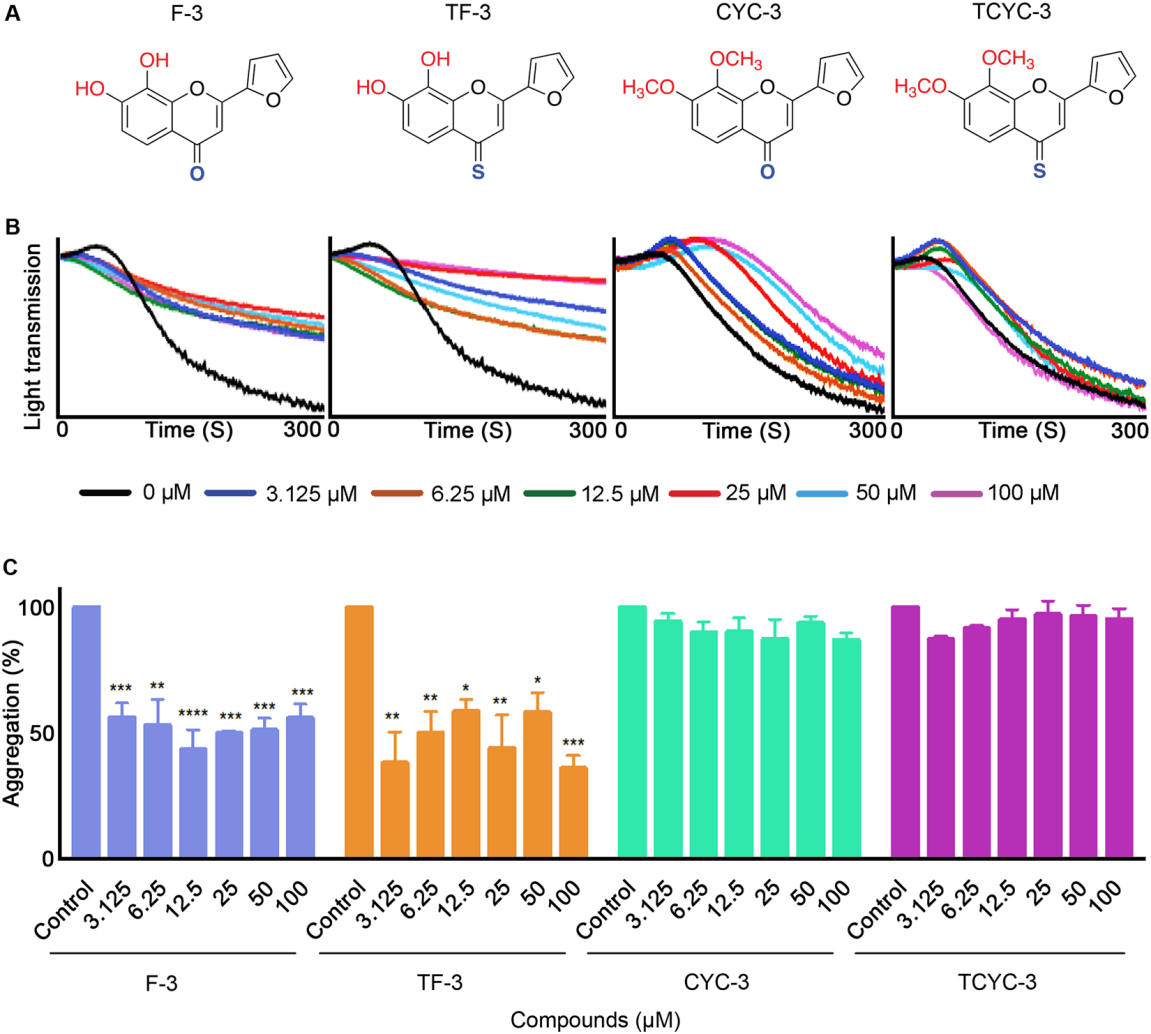
Effect of flavones with furan B-ring on human platelet activation. (**A**) Chemical structures of the flavones, F-3, TF-3, CYC-3 and TCYC-3. (**B**) Representative traces displaying the level of aggregation obtained when human isolated platelets were treated (for 5 minutes) with a vehicle control [0.1% (v/v) DMSO] or various concentrations of flavones, F-3, TF-3, CYC-3 and TCYC-3 (3.125–100 μM) and 0.5 μg/mL CRP-XL. (**C**) Bar graphs display the percentage of aggregation obtained in the presence and absence of diverse concentrations of flavones, F-3, TF-3, CYC-3 and TCYC-3. The data were normalised by considering the maximum aggregation observed for the vehicle control at 5 minutes as 100%, and the level of inhibition in flavone and its derivatives-treated platelet samples was calculated accordingly. Cumulative data denote mean ± S.D. (n = 3). The *p* values displayed (*p < 0.05, **p < 0.01, ***p < 0.001 and ****p < 0.001) are as analysed by one-way ANOVA using Graphpad Prism.

**Figure 4:**
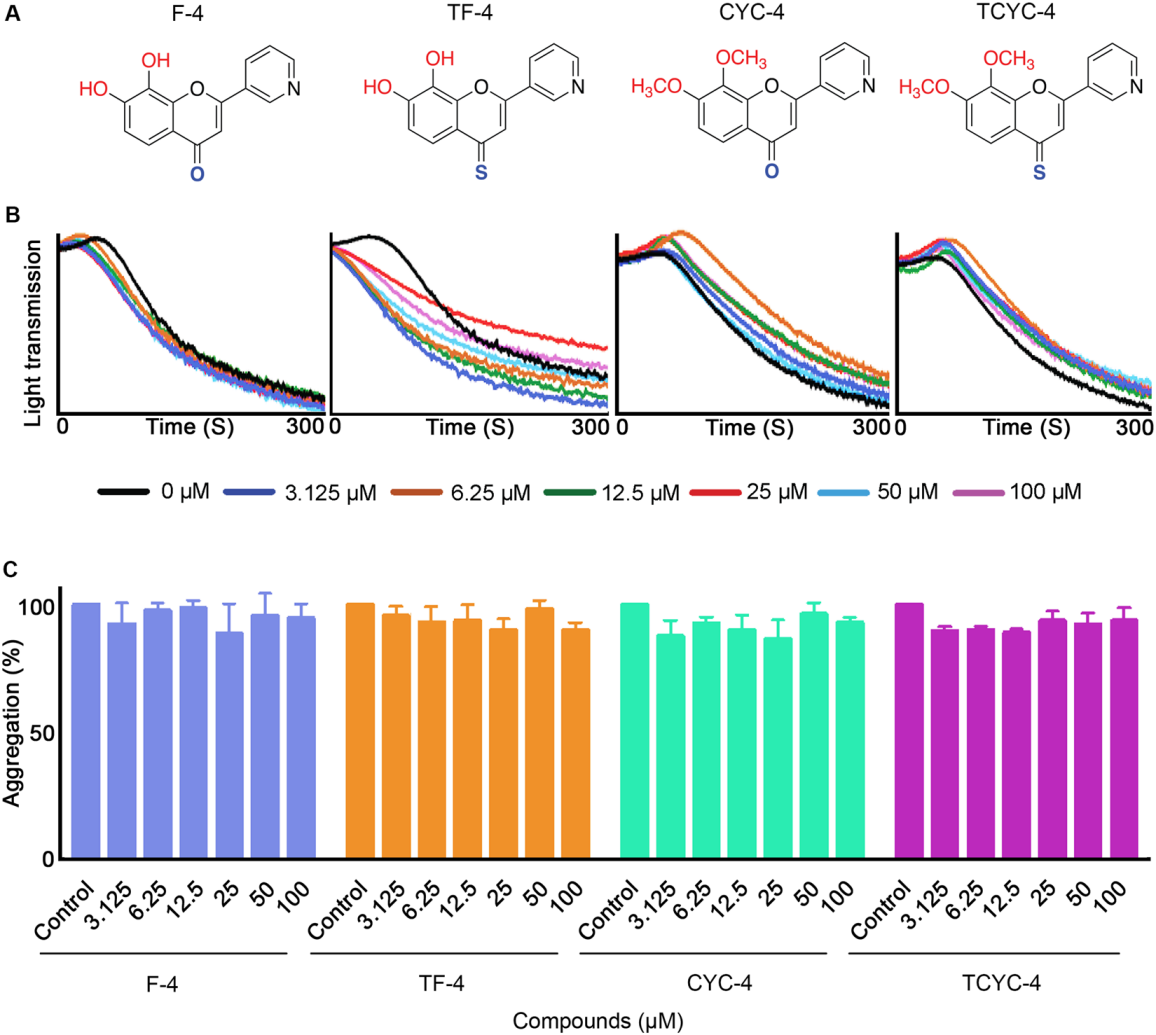
Effect of flavones with pyridyl B-ring on human platelet activation. (**A**) Chemical structures of the flavones, F-4, TF-4, CYC-4 and TCYC-4. (**B**) Representative traces showing the level of aggregation obtained when human isolated platelets treated (for 5 minutes) with a vehicle control [0.1% (v/v) DMSO] or various concentrations of flavones, F-4, TF-4, CYC-4 and TCYC-4 (3.125–100 μM) and 0.5 μg/mL CRP-XL. (**C**) Bar graphs show the percentage of aggregation obtained with flavones, F-4, TF-4, CYC-4 and TCYC-4. The data were normalised by considering the maximum aggregation observed for the vehicle control at 5 minutes as 100%, and the level of inhibition in flavone and its derivatives-treated platelet samples was calculated accordingly. Cumulative data denote mean ± S.D. (n = 3).

To determine the relationship between the specific functional groups in the structures of flavones and their antiplatelet activity, a series of hydroxy flavones, hydroxy 4-thioflavones, methoxy flavones and methoxy 4-thioflavones containing different B-ring (Figs 1A, 2A, 3A & 4A) were used in this study. These 16 flavones were synthesised based on the molecular template of 7,8-hydroxy flavone to systematically determine the influence of hydroxyl (-OH), methoxy (-OMe) and 4-thiocarbonyl (4-C=S) groups as well as the effects of phenyl group and its bioisosteres such as thiofuran, furanyl and pyridinyl moieties as B-ring on platelet activation/function. The synthesis of these compounds from our laboratories has been previously reported^35^ and the purities of compounds were analysed by reverse phase HPLC, and they were found to be >95%.

In order to determine the SAR of flavones with human platelets, the effects of 16 selected synthetic flavones on CRP-XL-stimulated platelet aggregation were evaluated by optical aggregometery. Human isolated platelets were treated with a vehicle [0.1% (v/v) DMSO] or diverse concentrations of flavones (3.125, 6.25, 12.5, 25, 50 and 100 μM) for 5 minutes prior to activation with 0.5 μg/mL CRP-XL for 5 minutes. None of the flavones exhibited activatory effects on platelets on their own, and the vehicle control containing 0.1% (v/v) DMSO did not affect platelet activation.

### Flavones with phenyl B-ring

Hydroxy flavone (F-1) with free hydroxyls and carbonyl moiety significantly inhibited CRP-XL-stimulated platelet aggregation at lower concentrations such as 3.125, 6.25 and 12.5 μM but no significant inhibition was observed at concentrations higher than 12.5 μM. The hydroxy 4-thioflavone (TF-1) showed significant inhibitory effects at all the concentrations tested. However, the methoxy flavone (CYC-1) inhibited the aggregation significantly only at 100 μM and the methoxy 4-thioflavone (TCYC-1) did not show any inhibitory effects at any of the concentrations tested (Fig. 1A–C).

### Flavones with thiofuran B-ring

Hydroxy flavone (F-2) displayed a similar inhibitory trend to F-1 with significant inhibition at lower concentrations up to 12.5 μM, whereas the hydroxy 4-thioflavone (TF-2) and the methoxy flavone (CYC-2) inhibited aggregation only at 100 μM. The methoxy 4-thioflavone (TCYC-2) of this group was found to possess no inhibitory activity on platelet aggregation (Fig. 2A–C).

### Flavones with furan B-ring

Hydroxy flavone (F-3) and hydroxy 4-thioflavone (TF-3) inhibited the CRP-XL-induced platelet activation at all the concentrations tested, however, the methoxy flavone (CYC-3) and the methoxy 4-thioflavone (TCYC-3) failed to inhibit the aggregation (Fig. 3A–C).

### Flavones with pyridine B-ring

None of the flavones with a pyridine B-ring displayed inhibitory effects on CRP-XL-stimulated platelet aggregation (Fig. 4A–C) at concentrations up to 100 μM.

It is interesting to note the impact of altering the B-ring on the platelet activity among the same class of flavones. For hydroxy flavones (with –OH and 4-C=O), changing the phenyl group (F-1) to a thiofuran group (F-2) did not affect the inhibitory potential as both compounds elicited the inhibitory activity up to 12.5 μM. Consistent inhibition (~65–70%) across the tested concentrations (3.125–100 μM) was observed when the phenyl group was replaced with a furan group (F-3). In contrast, substitution of the phenyl group with a pyridine group led to a complete abolition of inhibitory activity in platelets (F-4). A similar trend was observed for the hydroxy 4-thioflavones (with –OH and 4-C=S) when the B-ring phenyl group was replaced with its bioisosteres furan and pyridine. However, the substitution of thiofuran led to a reduction in the activity, for example, TF-2 showed ~50% inhibition only at 100 μM (Fig. 2) where its phenyl analogue, TF-1 elicited ~50% inhibition at all the concentrations used (Fig. 1).

The influence of B-ring modifications was not conspicuous among the methoxy flavones (with –OMe and 4-C=O) and methoxy 4-thioflavones (with –OMe and 4-C=S) as these two classes of flavones did not display any significant inhibitory activity on platelets in comparison to their hydroxyl analogues. Nevertheless, amongst the methoxy flavones, flavones with a B ring phenyl group (CYC-1) or thiofuran group (CYC-2) showed similar activities with inhibition only at 100 μM. Methoxy 4-thioflavones (with –OMe and 4-C=S) did not exert any inhibitory effects on platelets.

To corroborate the above results, the effects of these flavones on another platelet activation marker, specifically fibrinogen binding (a marker for inside-out signalling to integrin αIIbβ3), were measured by flow cytometry. The results obtained from this experiment concur with the aggregation data where hydroxy flavones F-1 (at 3.125–12.5 μM), F-2 (at 3.125–12.5 μM) and F-3 (at all concentrations tested), as well as thiohydroxy flavones TF-1 (at all concentrations tested), TF-2 (at 100 μM) and TF-3 (at all concentrations tested) significantly inhibited fibrinogen binding (Fig. S1), which is critical for subsequent platelet aggregation.

**Figure 5:**
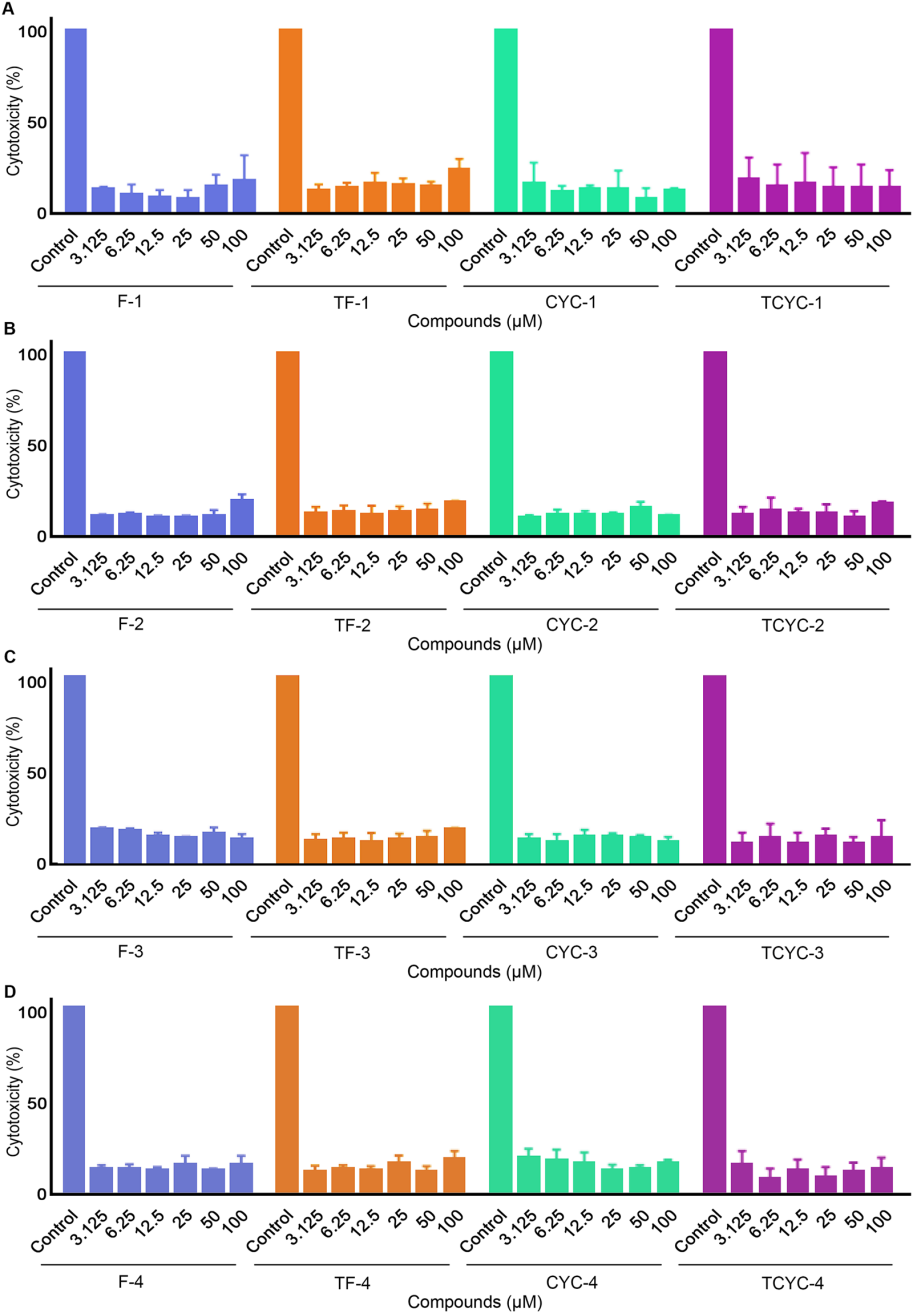
Cytotoxicity profile of flavones in human platelets. Human isolated platelets were treated with a positive control, or a vehicle control [0.1% (v/v) DMSO] or various concentrations (3.125–100 μM) of flavones, F-1, TF-1, CYC-1 and TCYC-1 (**A**), F-2, TF-2, CYC-2 and TCYC-2 (**B**), F-3, TF-3, CYC-3 and TCYC-3 (**C**) and F-4, TF-4, CYC-4 and TCYC-4 (**D**) for 30 minutes and the release of LDH, a marker for cytotoxicity was measured at 490 and 650 nm using spectrofluorimeter. The LDH release attained with the positive control was considered as 100%, and the levels of LDH release for flavone-treated samples were calculated. The data represent mean ± S.D. (n = 3). Statistical significance was analysed by one-way ANOVA using Graphpad Prism.

Finally, to determine whether the platelet inhibition observed was the result of a specific pharmacological effect of flavones rather than due to their cytotoxic effects, an LDH cytotoxicity assay was performed. For this, platelets were treated with a vehicle control [0.1% (v/v) DMSO] or diverse concentrations of flavones (3.125, 6.25, 12.5, 25, 50 and 100 μM) and the release of LDH, a cytosolic enzyme, which is an indicator of cellular toxicity was measured. As shown in Fig. 5, a positive control showed the maximum level of cytotoxicity with a higher LDH release, whereas the flavones did not exert cytotoxic effects at the concentrations (3.125–100 μM) used. These observations suggest that the inhibitory effects of flavones demonstrated in this study were not due to their cytotoxic effects on platelets.

## Discussion

Understanding the relationship between distinct functional groups within the structures of flavones and their influence on antiplatelet activity is critical for further development and modification of flavones in order to make them as more potent lead compounds for drug design. Such knowledge will aid in the development of improved therapeutic strategies for the prevention and treatment of cardiovascular disesaes, specifically thrombosis. In the present study, the analysis and comparison of the inhibitory activities of a series of 16 structurally-related synthetic flavones on CRP-XL-stimulated human platelet activation highlighted the key structural features that are required for the inhibition of platelet function.

In general, comparison between different classes of flavones with the same B-ring moiety suggested that the hydroxy flavones (with free -OH groups) were more effective than their corresponding methoxy flavones (with -OCH_3_ group). This highlighted that hydroxy groups, which are hydrogen bond donors, are essential for their inhibitory activities in platelets and that methoxy groups with hydrogen bond accepting profiles are less effective in this regard. However, the study by Bojić *et al*.^34^ reports increased anti-aggregatory potencies of *O*-methylated derivatives in comparison to their hydroxy analogues. This disparity could possibly be due to the difference in the hydroxyl substitution pattern of the flavones studied which would affect the interaction with the molecular target. In addition, several previous studies reported the loss of biological activity of flavonoids upon complete methylation of their active hydroxy groups^29,35,36^. Together with these previous studies, our data suggest that hydroxy groups are a key descriptor for the biological activity of flavonoids. It is worth highlighting that the hydroxy flavones used in this study possess hydroxyl groups at the C-7,8 position as opposed to the C-5,7 position in chrysin, a natural flavone that was previously reported to negatively modulate platelet activity^37^. When comparing the activity between chrysin and its 7,8-hydroxy analogue (F-1), it can be deduced that the position of the hydroxyl groups also influences platelet function as chrysin exhibited dose-dependent inhibition of platelet activity (6.25–100 μM)^38^, whereas, 7,8- hydroxyl flavone showed inhibition only between 3.25–12.5 μM. Further studies are required to determine the reasons for the low inhibitory effects obtained from higher concentrations of hydroxyl flavones with phenyl and thiofuran B-rings.

A number of previous studies have reported the significance of 4-C=O in the C-ring of flavonoids for antiplatelet activities based on the comparison between flavonoids with and without 4-C=O^32,34,39^. In this study, the influence of modification of 4-C=O to 4-C=S was also evaluated. Indeed, the replacement of 4-C=O with 4-C=S was well tolerated for flavones with free hydroxy groups (hydroxy flavones and hydroxy 4-thioflavones), however, no beneficial effects were observed for flavones with methoxy groups (methoxy flavones and methoxy 4-thioflavones). It is interesting to note that the influence of 4-C=S was also found to be dependent on the B-ring functionality as moderate loss of inhibitory activity was observed upon the replacement of 4-C=O with 4-C=S for flavones with a thiofuran B-ring. This demonstrates that the systematic analysis of flavones through careful correlation of effect of each substitution with respect to other functional groups is important for better optimisation of these compounds as molecular templates for drug design and discovery.

Natural flavonoids contain a phenyl group as the B-ring, hence, previous reports have focused on the influence of the position of the B-ring and its hydroxylation patterns. The present study involving synthetic flavonoids allowed the determination of the effect of incorporating bioisosteres of the phenyl group. It was found that replacing the phenyl group with a furan group was well tolerated for hydroxy flavones and hydroxy 4-thioflavones, whereas replacement of a phenyl group with a thiofuran group led to loss of inhibitory activities in platelets for hydroxy 4-thioflavones but was tolerated for hydroxy flavones. Conversely, replacement of the phenyl group with a pyridine group led to complete loss of inhibition in platelets. These observations suggest that the B-ring phenyl group is not critical for the antiplatelet activity, but the B-ring heteroatoms largely influence the activity. Furthermore, these observations suggest that the orientation and binding modes of the B-ring moieties might influence the interaction with their molecular targets. Hence, identification of the molecular targets for these flavones, and careful optimisation of the nature of the B-ring could lead to more efficacious flavone scaffolds for the development of novel antiplatelet agents. Furthermore, the hydroxy flavones and thiohydroxy flavones showed significant inhibitory effects on fibrinogen binding, a key marker for platelet activation via inside-out signalling to integrin αIIbβ3. This suggests that these flavones specifically with free hydroxy groups may modulate distinctive functions of platelets. The LDH cytotoxicity assay showed that these flavones are not cytotoxic to platelets at the concentrations tested and hence the inhibitory effects observed are due to their pharmacological effects on platelet function.

In conclusion, a panel of 16 structurally-related hydroxy flavones, methoxy flavones and their 4-thio analogues were screened for their antiplatelet activity upon CRP-XL-induced activation in human platelets. SAR analysis of these flavones suggested that the free hydroxyl group is essential for antiplatelet activity. Moreover, the modification at 4-C=O to 4-C=S in the C-ring, and B-ring modifications of phenyl group into specific bioisostere such as furanyl group, are well tolerated without any significant loss of their inhibitory activities. The molecular targets and the impact of these synthetic flavones on specific signalling pathways in platelets were not investigated in this study. Since the natural flavonoids posses broad spectrum of binding affinities and inhibitory activities against numerous cellular targets, the synthetic flavones with higher specificity for selective targets may be beneficial in achieving targeted effects. Therefore, further studies will be required to underpin the impact of these synthetic flavones with specific functional groups on various molecular targets in platelets. Together, the results obtained in this study with synthetic flavones enhance the current understanding of the SAR of flavones with human platelets and may aid in the design and development of novel anti-thrombotic strategies using flavones as potential molecular templates.

## Electronic supplementary material

Supplementary Information
