# Supplementary material for: Impact of specific functional groups in flavonoids on the modulation of platelet activation
Source: Sci Rep. 2018 Jun 22;8:9528. doi: 10.1038/s41598-018-27809-z (PMC6015034; doi:10.1038/s41598-018-27809-z)

**Impact of specific functional groups in flavonoids on the modulation of platelet activation**

Divyashree Ravishankar^1^, Maryam Salamah^1^, Angela Akimbaev^1^, Harry F. Williams^1^, Dina A. I. Albadawi^1^, Rajendran Vaiyapuri^2^, Francesca Greco^1^, Helen M.I. Osborn^1^, Sakthivel Vaiyapuri^1*^

^1^School of Pharmacy, University of Reading, Reading, UK

^2^School of Pharmacy, University of Reading Malaysia, Johar, Malaysia

**Corresponding Author**

^*^Sakthivel Vaiyapuri, School of Pharmacy, University of Reading, Reading, UK

Phone: +44 118 378 8015; E-mail: [s.vaiyapuri@reading.ac.uk](mailto:%73%2E%76a%69%79%61pur%69@rea%64%69ng.%61%63.u%6B)

Figure S1: **Effect of** **synthetic** **flavones on fibrinogen binding in human platelets**. Human isolated platelets were incubated with a vehicle control [0.1% (v/v) DMSO] or diverse concentrations of flavones, F-1, TF-1, CYC-1 and TCYC-1 **(A)**, F-2, TF-2, CYC-2 and TCYC-2 **(B)**, F-3, TF-3, CYC-3 and TCYC-3 **(C)** and F-4, TF-4, CYC-4 and TCYC-4 **(D)** for 5 minutes prior to stimulation with CRP-XL (0.5 μg/mL). This was followed by a further incubation of 20 minutes at room temperature. The level of fibrinogen binding (as a marker for inside-out signalling to integrin αIIbβ3) to the platelet surface was quantified using FITC-labelled anti-human fibrinogen antibodies by flow cytometry. The level of median fluorescence obtained with the vehicle control was taken as 100% to calculate the extent of inhibition in treated samples. The bar graphs display cumulative data as mean ± S.E.M (n=3). The *p* values shown (**p*<0.05, and ***p*<0.01) are as calculated by one-way ANOVA using Graphpad Prism.


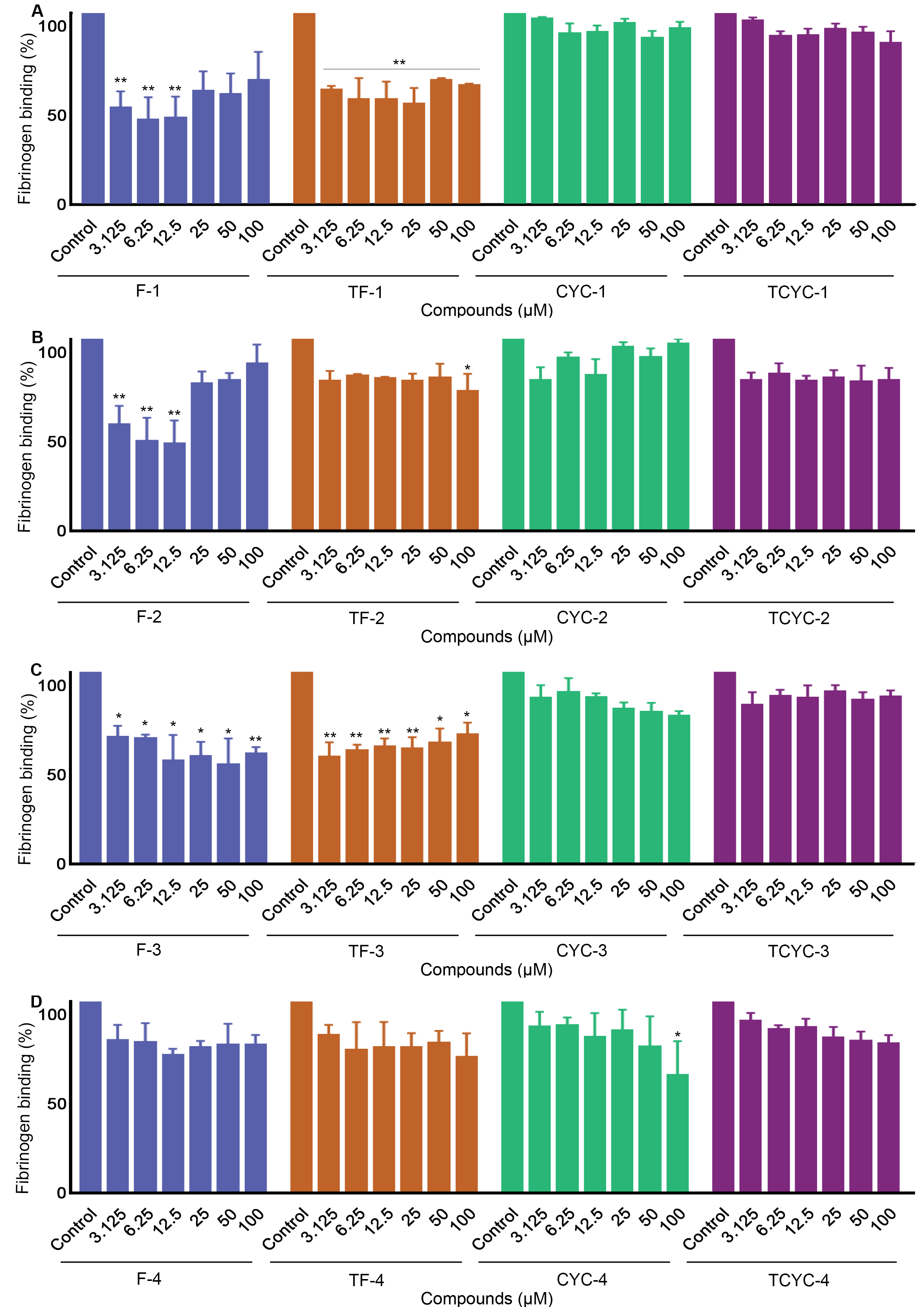

Supplement: Supplementary file 1 — Supplementary Information [file 41598_2018_27809_MOESM1_ESM.docx]
